# Proline Homeostasis in *Saccharomyces cerevisiae*: How Does the Stress-Responsive Transcription Factor Msn2 Play a Role?

**DOI:** 10.3389/fgene.2020.00438

**Published:** 2020-04-28

**Authors:** Noreen Suliani binti Mat Nanyan, Hiroshi Takagi

**Affiliations:** ^1^School of Industrial Technology, Universiti Sains Malaysia, Gelugor, Malaysia; ^2^Division of Biological Science, Graduate School of Science and Technology, Nara Institute of Science and Technology, Ikoma, Japan

**Keywords:** *Saccharomyces cerevisiae*, stress-responsive transcription factor, Msn2, proline homeostasis, proline permease, Gnp1, deubiquitinating enzymes, Ubp6

## Abstract

Overexpression of *MSN2*, which is the transcription factor gene in response to stress, is well-known to increase the tolerance of the yeast *Saccharomyces cerevisiae* cells to a wide variety of environmental stresses. Recent studies have found that the Msn2 is a feasible potential mediator of proline homeostasis in yeast. This result is based on the finding that overexpression of the *MSN2* gene exacerbates the cytotoxicity of yeast to various amino acid analogs whose uptake is increased by the active amino acid permeases localized on the plasma membrane as a result of a dysfunctional deubiquitination process. Increased understanding of the cellular responses induced by the Msn2-mediated proline incorporation will provide better comprehension of how cells respond to and counteract to different kinds of stimuli and will also contribute to the breeding of industrial yeast strains with increased productivity.

## Introduction

Cells of microorganisms are frequently exposed to a variety of stresses in their environment, such as prolonged nutrient starvation, free radicals and toxic molecules, imbalances in osmotic pressure and pH level, and non-optimal growth temperatures (Mager and De Kruijff, 1995; Ruis and Schüller, 1995). To survive in the face of such threatening and detrimental surroundings, eukaryotic cells have evolved a series of defense mechanisms at the transcriptional, protein, as well as metabolic levels (Boy-Marcotte et al., 1998; Toone and Jones, 1998; Estruch, 2000; Gasch et al., 2000; Kandror et al., 2004). External stimuli are perceived and transduced via the signal transduction pathways to cause global remodeling of gene expression, which is governed by transcriptional activators and repressors. In general, cellular stresses severely affect both transcription and translation activities, resulting in the inhibition of *de novo* protein synthesis. Moreover, environmental fluctuations may cause protein-related damages, such as the inhibition of enzyme activities, destabilization of cellular structures, and instability of chemical gradients, which eventually result in cell disruption. Thus, protein quality control and protein homeostasis are essential prerequisites for stress responses. Under harsh stresses, cells also undergo the systematic downregulation of energy-producing and energy-consuming processes in order to enter into a quiescent state, often accompanied by a dynamic shift in the central metabolic pathways that convert nutrients into energy and biomass. Cells possess tight and precise regulation systems to coordinate all the changes that are interconnected at those different levels.

In recent years, extensive research advances have been made in the field of stress responses using a eukaryotic model organism, the budding yeast *Saccharomyces cerevisiae* (Causton et al., 2001; Gasch, 2003). Earlier studies revealed the importance of the highly conserved stress-responsive transcription factors. Heat-shock factor 1 (Hsf1) was identified as a transcription activator that governs the expression of heat-shock proteins in response to elevated temperature (Sorger, 1990; Smith and Yaffe, 1991). The basic leucine-zipper transcription factor Yap1 is required for the induction of stress-responsive genes under oxidative stress conditions (Harshman et al., 1988; Moye-Rowley et al., 1989). Notably, *S. cerevisiae* cells have also developed species-specific transcription factors, namely Msn2 and Msn4 (Msn2/4) (Estruch and Carlson, 1993; Martínez-Pastor et al., 1996; Görner et al., 1998). Msn2/4 play pivotal roles in stress responses through the activation of hundreds of stress-related genes as a consequence to various stress conditions (Estruch, 2000; Gasch et al., 2000; Hasan et al., 2002; Berry and Gasch, 2008).

*S. cerevisiae* cells are also equipped with stress response mechanisms at the protein level to ensure protein quality at different subcellular locations, such as the cytosol (Hiraishi et al., 2009; Nillegoda et al., 2010; Theodoraki et al., 2012), endoplasmic reticulum (Brodsky, 2012; Thibault and Ng, 2012; Gardner et al., 2013; Wu et al., 2014), nucleus (Gardner et al., 2005; Rosenbaum and Gardner, 2011), mitochondria (Haynes and Ron, 2010; Baker and Haynes, 2011), and plasma membrane (Zhao et al., 2013; MacGurn, 2014; Shiga et al., 2014). The protein quality control includes all processes that ensure proper protein folding and thus prevent the toxic consequences of protein misfolding (Goldberg, 2003; Turcu et al., 2009). Irreversibly damaged proteins are selectively and effectively removed through proteasomal and/or vacuolar degradation systems, both of which consist of multiple fine-tuned steps including protein ubiquitination and deubiquitination (Finley et al., 2012).

Intracellular metabolism is dynamically changed in response to various stresses in *S. cerevisiae*, as well as in many other organisms. When the nutrient levels (e.g., carbon and nitrogen sources) are reduced, yeast cells reprogram the modes of energy metabolism from fermentation to respiration, a process termed a diauxic shift, in order to maximize the efficiency of energy production (Gray et al., 2004). Simultaneously, cells accumulate storage carbohydrates, such as trehalose and glycogen, which improve their survival rates under stress conditions and extend their life span (Fontana et al., 2010). In addition to carbon metabolites, nitrogen metabolites are consumed and produced in response to external stimuli. Recent studies have reported the significant importance of amino acids not only as building blocks of proteins but also in the control of cellular physiology (Sharma and Dietz, 2006; Takagi, 2008; Zhang et al., 2017). For instance, proline is accumulated and acts as an osmoprotectant in many plant and bacterial cells in response to osmotic stress (Csonka and Hanson, 1991; Kavi Kishor and Sreenivasulu, 2014). Besides that, glutamine plays a substantial role in mammalian cell growth where it facilitates the transport of other amino acids such as leucine into the cells and subsequently activates mTORC1 (Gonzalez and Hall, 2017). Collectively, these multiple regulatory mechanisms at the transcriptional, protein, and metabolic levels constitute a network that protects yeast cells from harmful conditions and allows them to adapt to new environments.

## Stress-Induced Gene Expression via Transcription Factors Msn2/4

The environmental stress response (ESR) controlled by the yeast-specific transcription factors Msn2 and Msn4 (Msn2/4) includes responses to various stresses, such as oxidative stress, osmotic shock, glucose starvation, high ethanol concentrations, temperature upshift, and freezing stress (Gasch et al., 2000; Izawa et al., 2007; Sadeh et al., 2011, 2012; Sasano et al., 2012a,b); together, these constituent responses of the ESR are required for both acute stress responses and cell survival during prolonged stress (Reiter et al., 2013). Although the Msn2/4 proteins were first reported to be 41% identical to each other and functionally redundant (Estruch and Carlson, 1993), subsequent studies demonstrated that Msn2 and Msn4 individually can induce the expression of different set of genes under certain stress conditions (Gasch, 2003; Watanabe et al., 2007; Berry and Gasch, 2008). Additionally, while the *MSN2* gene is constitutively expressed, transcription of the *MSN4* gene is induced by stress in an Msn2/4-dependent manner (Gasch et al., 2000). Thus, the roles of Msn2/4 are mostly overlapped but can be distinguished in part. Several studies suggest that Msn2 plays a role in transcriptional repression as well. The repression likely occurs via gene expression for transcription repressors or growth inhibitors. Msn2 activates the transcription of the *DOT1* gene, which encodes a repressor of the ribosome biogenesis gene (Elfving et al., 2014). Transcription of the *XBP1* gene, which encodes a repressor of cell-cycle associated genes, is also Msn2-dependent (Miles et al., 2013).

Under non-stress growth conditions, Msn2/4 are phosphorylated by cAMP-dependent protein kinase A (PKA) and reside in the cytoplasm. Once yeast cells are challenged by environmental perturbations, Msn2/4 are rapidly dephosphorylated and translocated into the nucleus (Görner et al., 1998; Beck and Hall, 1999). They then bind to the stress-response element sequence (STRE; AGGGG) in the promoter region of the target genes and subsequently activate the transcription (Boy-Marcotte et al., 1998, 1999; Gasch et al., 2000; Causton et al., 2001). Previous studies identified functional domains of Msn2, which include the C-terminal zinc finger DNA-binding domain (DBD) (Marchler et al., 1993; Martínez-Pastor et al., 1996; Schmitt and McEntee, 1996; Moskvina et al., 1998), the nuclear localization signal (NLS) region (Görner et al., 1998, 2002), the nuclear export signal (NES) region (Görner et al., 1998), and the imperative transcriptional activating domain (TAD) at the N terminus (Boy-Marcotte et al., 2006). In addition to phosphorylation by PKA, multiple upstream pathways are involved in the regulation of Msn2 and/or Msn4: the target-of-rapamycin (TOR) signaling-dependent cytoplasmic localization (Beck and Hall, 1999), the karyopherin Msn5-dependent nuclear export (Chi et al., 2001; Görner et al., 2002), proteasome-mediated degradation (Durchschlag et al., 2004), the ubiquitin ligase Rsp5-dependent nuclear export of mRNA (Haitani and Takagi, 2008), and the protein kinase Rim15-dependent phosphorylation (Lee et al., 2013).

To understand how Msn2/4 contribute to stress responses, the downstream target genes of Msn2/4 have been comprehensively investigated. First, Msn2/4 directly induce the expression of the genes encoding antioxidant enzymes, such as *CTT1* (for catalase), *SOD1* and *SOD2* (for superoxide dismutases), and *PRX1* and *TSA2* (for thiol peroxidases) (Hasan et al., 2002; Drakulic et al., 2005; Sadeh et al., 2011). Since various kinds of stresses lead to the imbalanced generation of reactive oxygen species (ROS) causing cell death, the elimination of ROS by the antioxidant enzymes is an important stress response. Second, Msn2/4 are essential to the induction of the genes involved in protein quality control, such as the molecular chaperone gene *HSP12*, the sHSP-family genes *HSP26* and *HSP42*, the HSP70-family genes *SSA1* and *SSA4*, and the HSP90-family genes *HSP82* and *HSP104* (Kandror et al., 2004; Eastmond and Nelson, 2006). Under stress conditions, the expression of the polyubiquitin precursor gene *UBI4* is also upregulated to mark proteins for selective degradation via the ubiquitin-proteasome system (Simon et al., 1999). Third, Msn2/4 triggers metabolic reprogramming in response to stress by inducing the expression of the mitochondrial respiratory genes *COX5b, COX17*, and *COX20*, the pentose phosphate pathway genes *SOL4, GND2*, and *TKL2*, the trehalose synthetic genes *TPS1, TPS2, TPS3*, and *TSL1*, and the glycogen synthetic genes *GSY1, GSY2*, and *GLC3* (Estruch, 2000; Gasch et al., 2000; Causton et al., 2001; Sadeh et al., 2011).

Overexpression of *MSN2* or *MSN4* has been a promising approach for the construction of industrial yeast strains to improve their stress resistance as well as fermentation ability (Cardona et al., 2007; Watanabe et al., 2009; Sasano et al., 2012a,d). For instance, baker's yeast cells are challenged by a variety of baking-associated stresses during dough fermentation, including freeze-thaw, air-drying, and a high sugar content, which trigger the oxidative stress due to the accumulation of intracellular ROS caused by protein misfolding and mitochondrial damage (Kitagaki and Takagi, 2014). Our previous study reported that baker's yeast cells that overexpressed *MSN2* have shown a higher tolerance to freezing stress and enhanced the ability of yeast cells to ferment productively in frozen dough (Sasano et al., 2012a). Furthermore, in second-generation bioethanol production with lignocellulosic biomass, several growth/fermentation inhibitors such as furfural and 5-hydroxymethylfurfural are generated and they are known to produce ROS (Allen et al., 2010). The overexpression of *MSN2* in bioethanol yeast strains that were grown in the presence of furfural upregulates the transcription of antioxidant gene, which in turn increases the cell resistance and leads to recovery of cell growth (Sasano et al., 2012d). Our recent study also showed that the overexpression of *MSN2* shortens the replicative lifespan of yeast cells by reducing the intracellular proline levels (Mukai et al., 2019).

## Amino Acids Involved in Stress Response

Among major carbon and nitrogen metabolites, Takagi's laboratory has focused on amino acids as hallmarks and mediators of *S. cerevisiae* stress responses (Takagi et al., 2000; Morita et al., 2002; Matsuura and Takagi, 2005; Kaino et al., 2008; Takagi, 2008; Nishimura et al., 2010). Amino acids essentially serve as a nitrogen source and the building blocks of proteins in yeast. Furthermore, they also contribute to the proliferation and durability of yeast cells following exposure to stresses. For instance, proline and charged amino acids such as arginine and glutamate were suggested to be pivotal for cell resistance in yeast and *Escherichia coli* cells, as these amino acids can inhibit the denaturation of proteins (Takagi et al., 1997; Morita et al., 2002; Shiraki et al., 2002; Chattopadhyay et al., 2004; Golovanov et al., 2004).

In terms of stress-resistance activity, proline is one of the most studied among the 20 naturally occurring amino acids. It has a cryoprotective activity in *S. cerevisiae* cells, as well as in many other kinds of cells (Sleator and Hill, 2001; Maggio et al., 2002; Krishnan et al., 2008; Liang et al., 2013). Although proline synthesis is not induced in response to stress, intracellular proline accumulation via the engineering or modification of the enzymes involved in the proline synthesis and degradation pathway in industrial baker's yeast strains elevates the resistance of freeze-thaw stress, leading to an enhanced fermentation ability in frozen dough (Kaino et al., 2008; Sasano et al., 2012c; Tsolmonbaatar et al., 2016). In addition to the freeze-thaw stress tolerance, proline confers tolerance to high osmolality, desiccation, high concentrations of ethanol, and weak acids (Takagi et al., 2000, 2016; Sasano et al., 2012b). Although the involvement of proline carries considerable importance in general stress responses, the roles played by Msn2/4 that link to the proline homeostasis is poorly understood.

The concerted processes of biosynthesis, degradation, and transport of proline administer the cellular proline homeostasis. Proline, which is incorporated into proteins, is synthesized from glutamate in three enzymatically catalyzed steps. First, the γ-glutamyl kinase Pro1 catalyzes the conversion of glutamate to glutamate-5-phosphate (Brandriss, 1979). Then, the unstable glutamate-5-phosphate is converted to glutamate semialdehyde by the γ-glutamyl phosphate reductase Pro2 (Tomenchok and Brandriss, 1987). Glutamate semialdehyde, then spontaneously cyclizes to form Δ^1^-pyrroline-5-carboxylate (P5C), which is converted to proline by the P5C reductase Pro3 (Brandriss and Falvey, 1992). Pro1 is sensitive to proline feedback inhibition, and thus, several known amino acid changes, such as Ile150Thr and Asp154Asn, in Pro1 alleviate feedback inhibition and elevate the level of intracellular proline (Morita et al., 2003; Sekine et al., 2007). At the transcriptional level, only the expression of the *PRO2* gene is under the general amino acid control system (Natarajan et al., 2001), and it is still unknown whether the proline-synthetic pathway genes are coordinately transcribed by a certain external stimulus. To assimilate proline as a nitrogen source, it is degraded into glutamate via the proline oxidase Put1 and the P5C dehydrogenase Put2, both of which are mitochondrial enzymes (Brandriss, 1979). Loss of the Put1 function contributes to an increase of the intracellular proline content (Takagi et al., 2000). Nitrogen catabolite repression (NCR) transcriptionally represses both the *PUT1* and *PUT2* genes (Hofman-Bang, 1999; Georis et al., 2009), which are positively regulated by the transcription activator Put3 (Ann et al., 1996). NCR prevents the utilization of proline as a nitrogen source when rich nitrogen compounds, such as ammonia and glutamine, are present.

In *S. cerevisiae*, the amino-acid-polyamine-organocation (APC) superfamily consists of 24 permease proteins whose function is to transport amino acids and other amines into the cells (Nelissen et al., 1997; Jack et al., 2000). Four of them, namely Gap1, Put4, Agp1, and Gnp1 are responsible to incorporate proline (Andréasson et al., 2004). Gap1 encodes a high capacity transporter for all naturally occurring amino acids and is regulated by the quality of the nitrogen source present in the growth medium (Grenson et al., 1970; Chen and Kaiser, 2002). Put4 is required for the high-affinity transport of proline and is regulated at the transcriptional level by NCR (Xu et al., 1995; Ter Schure et al., 2000). On the other hand, Agp1 and Gnp1 encode permeases with broad substrate specificity and a high affinity for glutamine, respectively (Zhu et al., 1996; Iraqui et al., 1999). The *AGP1* and *GNP1* genes are induced by the regulation of the Ssy1-Ptr3-Ssy5 amino acid sensor complex (Didion et al., 1998; Iraqui et al., 1999; Forsberg et al., 2001; Ljungdahl, 2009).

Structural analogs of amino acids have been widely used to analyze amino acid homeostasis. l-azetidine-2-carboxylic acid (AZC), a toxic analog for proline, is used in both fundamental and applied researches, as it has been proven to be beneficial to study the cellular metabolism and the production of macromolecules in both prokaryotes and eukaryotes (Bach and Takagi, 2013). AZC is a non-protein amino acid originally found in plants and has a heterocyclic structure with a four-membered nitrogen ring and a carboxylic acid group on one of the ring carbon atoms. The main difference between AZC and proline is that the former has a four-ring member while the latter has a five-ring member. AZC, as well as many other amino acid analogs, is thought to be toxic to cells, because it is carried into the cells through proline permeases, and competes with proline during incorporation into nascent proteins, which consequently causes protein misfolding and cell death (Trotter et al., 2001; Weids and Grant, 2014).

## Prospective Roles of *MSN2* Overexpression in the Control of Proline Homeostasis

Our recent study found that overexpression of *MSN2* increased the sensitivity of yeast cells to several toxic amino acid analogs, namely AZC, *o*-fluoro-dl-phenylalanine (OFP), and l-canavanine (Can), which are known to be the analogs of proline, phenylalanine, and arginine, respectively (Mat Nanyan et al., 2019a). This suggests that *MSN2* overexpression negatively controls the growth and survival of yeast cells in media containing those toxic compounds, indicating that overexpression of *MSN2* is involved in the incorporation of amino acids into the cells (Mat Nanyan et al., 2019a). Further investigation showed that the increased AZC sensitivity in *MSN2*-overexpressing (*MSN2*-OE) cells could be due to the increased incorporation of AZC, since higher AZC levels were detected in *MSN2*-OE cells than that observed in wild-type cells (Mat Nanyan et al., 2019a). Not only AZC, but also the overexpression of *MSN2* increased proline levels shortly after the addition of proline into the culture (Mat Nanyan et al., 2019a). Our study also found that quadruple disruption of proline permease genes (*GAP1, PUT4, AGP1*, and *GNP1*) in *MSN2*-OE cells with the strain CAY29 background conferred higher resistance to AZC (Mat Nanyan et al., 2019a), similar to that observed in wild-type cells with the same quadruple disruption (Andréasson et al., 2004). Moreover, a single disruption of *GNP1* showed the most striking effect among single deletions of the permease genes, where Δ*gnp1* cells showed a higher resistance against AZC, highlighting a predominant role played by Gnp1 in the AZC incorporation as compared to the other three permeases, which suggest that they may have redundant roles (Mat Nanyan et al., 2019a). Consistently, the overexpression or deletion of *GNP1* has no significant effect on the growth of yeast when grown in the media containing OFP or Can because Gnp1 does not incorporate phenylalanine (transported by Gap1, Agp1, and Bap2) or arginine (transported by Can1; Ljungdahl and Daignan-Fornier, 2012). Intriguingly, although the transcription of the typical Msn2-targeted gene *CTT1* was highly upregulated under *MSN2* overexpression, none of the levels of proline permease gene mRNA transcripts were significantly upregulated in *MSN2*-OE cells compared to that of in the wild-type cells, which signifies that *GNP1* or other proline permease genes are not transcriptionally activated by Msn2 (Mat Nanyan et al., 2019a). More importantly, the Gnp1-GFP signal is highly detected in *MSN2*-OE cells, which is largely distributed on the plasma membrane rather than other parts of the cells (Mat Nanyan et al., 2019a). Thus, these results indicate that the endocytic degradation of Gnp1 was defective under *MSN2* overexpression, which highlights the newly discovered network between the ESR which is mediated by Msn2 and proline homeostasis in yeast. It would be intriguing to further examine whether proline permeases and intracellular proline homeostasis under different stress conditions are enhanced via the blocking of endocytosis.

**Figure 1:**
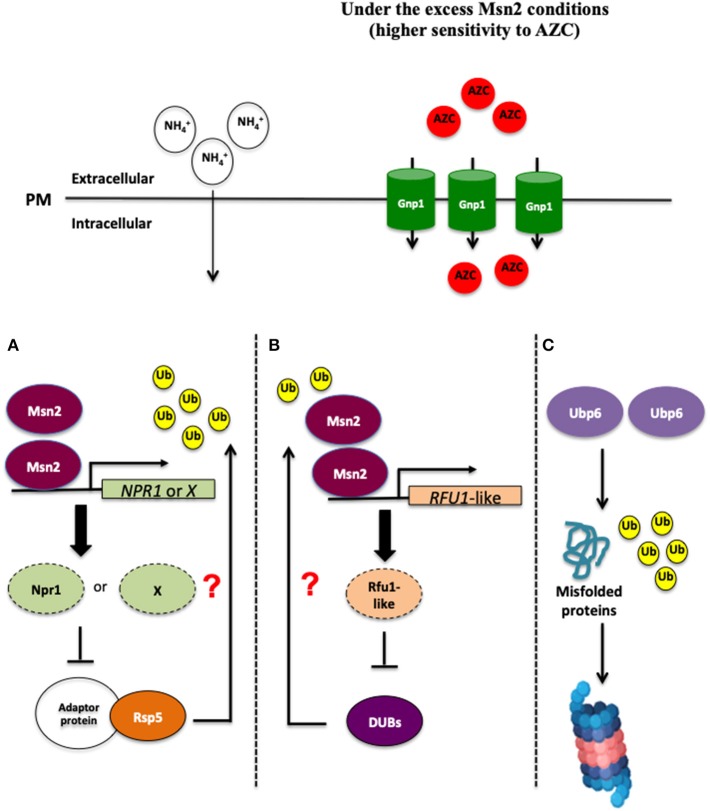
An excess of Msn2 inhibits the endocytic degradation of Gnp1 by unknown mechanisms. There are at least three plausible mechanisms, which could explain this phenomenon. **(A)** Overexpression of *MSN2* might induce the expression of *NPR1* or the associated inhibitor genes for Rsp5-independent protein ubiquitination, therefore Gnp1 could not be ubiquitinated and endocytosed. **(B)** Secondly, a high level of Msn2 may activate the gene expression of DUB repressors such as Rfu1-like protein(s), leading to a reduced or loss function of DUBs. **(C)** In yeast cells overexpressing *UBP6*, an excess of Ubp6 may enhance the ubiquitination and accelerate the degradation of misfolded proteins by unknown mechanisms, for example, the proteasome-mediated mechanism in the *MSN2*-independent manner, leading to the tolerance toward proteotoxic stress caused by intracelullar accumulation of the misfolded proteins.

Ubiquitination and deubiquitination are involved in the control of endocytic degradation of amino acid permeases localized on the plasma membrane (Springael and André, 1998; Kimura et al., 2009; Kimura and Tanaka, 2010; Jones et al., 2012; MacGurn, 2014). Since the E3 ubiquitin ligase Rsp5 is responsible for regulating the localization of Gnp1 (Sasaki and Takagi, 2013), *MSN2* overexpression might negatively control the protein ubiquitination mediated by Rsp5. The expression of the kinase gene *NPR1* or other associated inhibitor genes for Rsp5-dependent protein ubiquitination might be induced as a result of *MSN2* overexpression, and could thereby inhibit the endocytosis of Gnp1 (Figure 1). Although it is still hard to predict the target of Msn2 that is responsible for the endocytosis of Gnp1, it is crucial to explore and determine the target of many regulators, including the TORC1 pathway, Rsp5, and endocytosis. Interestingly, our recent study demonstrated that yeast cells overexpressing *MSN2* exhibited a higher level of ubiquitinated proteins and a shortage of free ubiquitin content (Mat Nanyan et al., 2019b), similar to what is observed in Δ*ubp6* cells, where the absence of *UBP6* gene encoding one of the yeast deubiquitinating enzymes (DUBs) and is classified into ubiquitin-specific-protease (USP) family caused depletion of free cellular ubiquitin (Chernova et al., 2003; Hanna et al., 2003). These results suggest that the excess level of Msn2 reduces or impairs the activity of DUBs, which could result in the inhibition of endocytosis of Gnp1 (Mat Nanyan et al., 2019b). Further investigations showed that the transcription of *UBP6* and other DUB genes was not significantly changed in *MSN2*-OE cells, indicating that Msn2 does not repress *UBP6* and other DUB genes transcriptionally (Mat Nanyan et al., 2019b). The disruption of *UBP6* in yeast cells makes them more susceptible to the toxicity of translational inhibitors such as cycloheximide and the toxic arginine analog, Can, which are caused by a deficiency of free ubiquitin (Chernova et al., 2003; Hanna et al., 2003). Intriguingly, deletion of *UBP6, UBP3* as well as *OTU1* genes aggravates the growth inhibitory effect of toxic amino acid analogs in yeast cells (Mat Nanyan et al., 2019b). In particular, disruption of *UBP6* led to the most remarkable sensitivity toward AZC, OFP, and Can (Mat Nanyan et al., 2019b). These phenomena were also observed in *MSN2*-OE cells, suggesting that the excess level of Msn2 and Ubp6 deficiency confer a common phenotype of defective resistance against toxic amino acid analogs. The combination of the *MSN2* overexpression and *UBP6* disruption resulted in growth similar to that seen with Δ*ubp6* cells in the presence of low concentrations of AZC (Mat Nanyan et al., 2019b). Moreover, the co-overexpression of *MSN2* and *UBP6* increased the resistance to AZC, OFP, and Can (Mat Nanyan et al., 2019b). Further investigations should be carried out to clarify whether Msn2 and Ubp6 affect AZC resistance via the same mechanism or whether Ubp6 functions independently to counteract the toxicity of amino acid analogs (Figure 1). Intriguingly, the Gnp1-GFP signals were elevated in Δ*ubp6* cells and were mostly localized on the plasma membrane, similar to what was observed in *MSN2*-OE cells (Mat Nanyan et al., 2019b). The impaired deubiquitination mediated by an excess of Msn2 might cause the inhibition of the endocytic degradation of proline permeases by unknown mechanisms. We propose here a novel role of Msn2 in the control of intracellular uptake of proline (Figure 1). Msn2 is known for its global effect in the control of various regulatory networks in the face of stress conditions such as regulating the antioxidant enzyme genes and molecular chaperones, as well as reprogramming the carbon metabolism. In addition to that, the novel link suggested between Msn2 and proline uptake might further contribute to a deeper comprehension of global stress responses in *S. cerevisiae* in order to withstand various fluctuating growth conditions.

## Conclusion

In this mini-review, we discuss the current understanding of the stress-responsive transcription factor Msn2 and the stress response in yeast. In particular, the overexpression of *MSN2* has been shown to increase the tolerance of yeast cells for various kinds of stress conditions, such as oxidative and freezing stresses. In addition, we shed light on a potentially important link, namely that the inhibition of endocytic degradation mediated by Msn2 plays an essential role in regulating the proline homeostasis under stress conditions, evidently caused by the loss of function of DUBs. Further investigations will elucidate the profound relationship among Msn2, DUBs, and Gnp1 in the regulation of proline homeostasis, which may serve as a foundation to engineer more robust industrial yeast strains, which might be advantageous in fermentation industries.

## Author Contributions

NM and HT substantially and intellectually contribute to this work, and reviewed and approved the final version of manuscript.

## Conflict of Interest

The authors declare that the research was conducted in the absence of any commercial or financial relationships that could be construed as a potential conflict of interest.
